# Likely a matter of time for Amyloid-β immunotherapy

**DOI:** 10.1038/s43856-021-00010-6

**Published:** 2021-06-30

**Authors:** Andreia Cunha

**Affiliations:** Communications Medicine, http://www.nature.com/commsmed

## Abstract

Amyloid-β peptide (Aβ) deposition in the brain is an early feature of Alzheimers’ disease. In a phase II clinical trial recently published in *The New England Journal of Medicine*, Mintun and colleagues report on the safety and efficacy of an antibody targeting Aβ peptide in amyloid plaques for the treatment of participants with early symptomatic Alzheimer’s disease.

**Figure:**
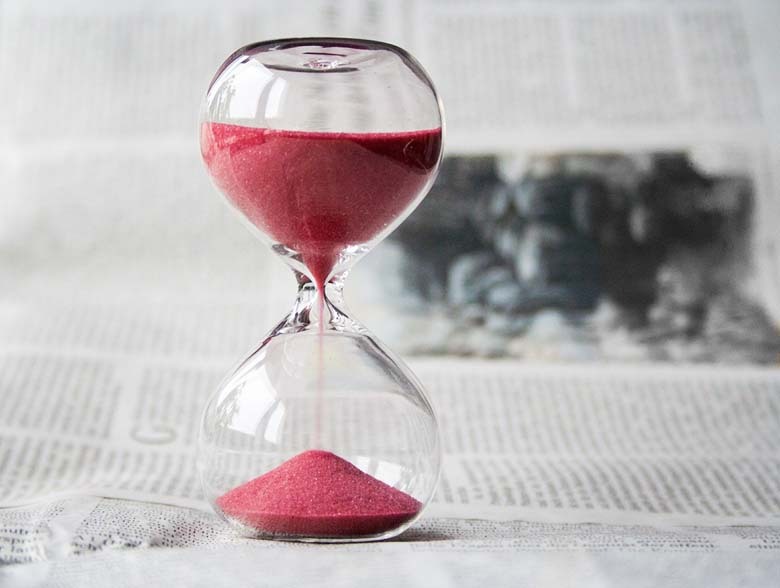
Pixabay

According to WHO, nearly 50 million people worldwide live with dementia, and the majority of these have Alzheimer’s disease. There are currently no therapies to stop or reverse the disease trajectory, only to ameliorate symptoms, and delay the cognitive decline characteristic of Alzheimer’s disease.

Therapies targeting amyloid plaques have received a lot of attention over the years, but with clinical trials being largely unsuccessful, it remains uncertain how fruitful this approach might be. The US Food and Drug Administration approved this month a monoclonal antibody targeting Aβ, aducanumab, for the treatment of Alzheimer’s disease, for the first time, but the potential clinical benefit remains uncertain. Among other considerations, it is thought that time is of the essence for therapies targeting Aβ, since its deposition in the brain can begin many years before symptoms develop.

Mintun and colleagues focused on individuals early in the Alzheimer’s disease trajectory and performed a multicentre, randomised, double-blind, placebo-controlled phase II trial (TRAILBLAZER-ALZ) that assessed the safety and efficacy of donanemab, another monoclonal antibody targeting Aβ in amyloid plaques^1^. Participants had mild symptoms and detectable tau and amyloid deposition by positron-emission tomography (PET) below a critical threshold of advanced disease.

A total of 257 patients were randomised 1:1 to either intravenous donanemab or placebo, given every 4 weeks up to 72 weeks. The primary outcome was the change from baseline in the score on the Integrated Alzheimer’s Disease Rating Scale (IADRs), a scale from 0 to 144, in which the lower the score the higher the cognitive and functional impairment. Secondary outcomes included assessment of clinical dementia severity, cognitive and functional abilities, and change in the amyloid and tau burden on PET.

Both groups had the same iADRS score at baseline, but at 76 weeks the group that received donanemab had a change from baseline of −6.86 while that of the placebo group was −10.06, a modest 3.20 point difference that was statistically significant, albeit marginally. At 76 weeks, amyloid plaque level was reduced to normal in 68% of the participants that received donanemab but this was not correlated with individual clinical outcomes. The remaining secondary outcomes showed mostly no difference between the two groups.

In terms of safety, both groups were similar in the number of deaths or adverse events and at least one adverse event was experienced by 90% of participants in each group. However, the incidence of amyloid-related cerebral edema or effusions was higher in the donanemab group.

This report is encouraging and brings a cautious optimism to the field of Aβ immunotherapies, underscoring the potential importance of targeting Aβ as early as possible. However, additional trials will be necessary to get a clearer picture of the clinical potential of donanemab.

## References

[1] Mintun MA (2021). Donanemab in Early Alzheimer’s Disease.. N. Engl. J. Med..

